# Case report of Lewy body disease mimicking Creutzfeldt-Jakob disease in a 44-year-old man

**DOI:** 10.1186/s12883-016-0643-y

**Published:** 2016-07-30

**Authors:** Laure Saint-Aubert, Jérémie Pariente, Herve Dumas, Pierre Payoux, Jean-Philippe Brandel, Michèle Puel, Anne Vital, Eric Guedj, Suzanne Lesage, Katell Peoc’h, Christine Brefel Courbon, Fabienne Ory Magne

**Affiliations:** 1 Department NVS, Center for Alzheimer Research, Translational Alzheimer Neurobiology, Karolinska Institutet, Stockholm, Sweden; 2 INSERM UMR825, Imagerie cérébrale et handicaps neurologiques, Toulouse, France; 3 University of Toulouse, Imagerie cérébrale et handicaps neurologiques, Toulouse, France; 4 Department of Neurology, University Hospital of Toulouse, Toulouse, France; 5 Department of Neuroradiology, University Hospital of Toulouse, Toulouse, France; 6 Department of Nuclear Medicine, University Hospital of Toulouse, Toulouse, France; 7 AP-HP, Cellule Nationale de Référence des Maladies de Creutzfeldt-Jakob, Groupe Hospitalier Pitié-Salpêtrière; Inserm U 1127, CNRS UMR 7225, Sorbonne Universités, UPMC Univ. Paris 06 UMR S 1127, Institut du Cerveau et de la Moelle épinière, ICM, 75013 Paris, France; 8 Department of Pathology, University Hospital of Bordeaux, Bordeaux, France; 9 Aix-Marseille University, CNRS, UMR7289, INT, 13005 Marseille, France; 10 Sorbonne Universities, UPMC (Paris 6), Inserm U1127, CNRS UMR 7225, and ICM, Paris, France; 11 Department of Biochemistry, APHP, Lariboisière Hospital, Paris, France; 12 Department of Clinical Pharmacology, University of Medicine, Toulouse, France

**Keywords:** Dementia with Lewy bodies, 14–3–3 protein, Diagnosis, Genetics, Imaging

## Abstract

**Background:**

Few patients are reported with dementia with Lewy bodies before fifty years-old, which may partly reflect the difficulty of accurate diagnosis in young population. We report the case of a 44-year-old male with pathologically confirmed sporadic dementia with Lewy bodies, who did not fulfil the revised clinical criteria for this disease.

**Case presentation:**

We document this atypical case with clinical and cognitive evaluation, imaging, biochemistry, genetics and pathology investigations. Creutzfeldt-Jakob disease was first suspected in this patient with no previous medical history, who developed acute and rapid cognitive impairment, L-dopa-non-responsive parkinsonism, and delusion. Positive 14–3–3 protein was initially detected in cerebrospinal fluid and until the late stages of the disease. Severe atrophy with no diffusion hypersignal was found on structural MRI as well as an extensive hypometabolism on ^18^F-FDG-PET, in comparison to age-matched healthy volunteers. Genetic investigation found no alpha-synuclein gene mutation. The patient died within 5 years, and post-mortem examination found numerous Lewy bodies and Lewy neurites consistent with pure Lewy body disease.

**Conclusions:**

This comprehensively described case illustrates that dementia with Lewy bodies can occur in young patients with atypical clinical presentation. Biochemistry and neuroimaging investigations can sometimes be insufficient to allow accurate diagnostic. More specific markers to support such diagnosis are needed.

**Electronic supplementary material:**

The online version of this article (doi:10.1186/s12883-016-0643-y) contains supplementary material, which is available to authorized users.

## Background

Dementia with Lewy bodies (DLB) is the second most common form of neurodegenerative dementia in the population over 65 years-old [1], with an age of onset varying from 50 to 80 [2]. The prevalence of DLB in younger patients remains unknown. The recent criteria for diagnosis of probable DLB require progressive cognitive decline, accompanied by two out of three core features: fluctuating alertness, recurrent visual hallucinations and spontaneous motor Parkinsonism [3]. Despite these criteria, clinical diagnosis of DLB may be difficult, and some cases may be misdiagnosed with other dementia such as Creutzfeldt-Jakob disease (CJD) [4]. Indeed, CJD cases may share clinical similarities with DLB such as dementia, visual disturbances and abnormal movements (parkinsonism, myoclonia, dystonia and chorea). Nevertheless, the onset of the symptoms in CJD is usually acute while insidious in DLB. We report the case of a 44 year-old patient with acute dementia, first mimicking CJD, but proved to have DLB at autopsy. Clinical, neuropsychological, imaging and biological investigations are reported.

## Case presentation

### Clinical follow-up

- Month 0: A 44 year-old man, 12 years of education, started complaining acutely of slowness and sustained attention difficulties. He had neither clinical comorbidities nor relevant familial history.

Within three weeks after the first complaints, his status rapidly worsened and the patient needed help for every daily life activities. He was hospitalized, and presented significant cognitive impairment and walking difficulties related to marked parkinsonian syndrome. Depression with suicidal ideation was reported. No cerebellar features were observed. Neuropsychological assessment showed marked cognitive slowness and impairment of anterograde memory, mental calculation, verbal fluency, praxies and mental flexibility (Table 1). Electroencephalogram (EEG) showed slow cerebral activity without any epileptic or pseudo periodic abnormalities. Tianeptine and ropinirole treatments were introduced.

**Table 1:** Clinical, neuropsychological and biological assessments for the patient

		M0	M2	M7	M15	M27	M43
**Neuropsychological assessment**							
	**Global cognitive state**						
	MMSE	25	25	24	21	17	13
	**Speed processing**						
	TMT part A, time (seconds)	133	157	144	193	NA	NA
	**Short term memory**						
	WAIS III digit span forward	4	5	5	5	3	NA
	WAIS III digit span backward	3	3	2	3	2	NA
	**Executive functions**						
	Phonemic verbal fluency: letter ‘p’	12	1	6	10	NA	NA
	Semantic verbal fluency: ‘animal’ category	15	16	16	12	NA	NA
	FAB (/18)	12	12	12	10	6	2
	TMT part B, time (seconds)	296	537	486	NA	NA	NA
	TMT part B, mistakes	0	1 (help)	1			
	**Anterograde verbal memory**						
	FCSRT						
	sum of free recalls (/48)	19	18	27	22	8	NA
	sum of free + cued recalls (/48)	42	47	46	45	NA	NA
	recognition (/48)	47	48	48	45	NA	NA
	delayed free recall (/16)	9	6	8	6	NA	NA
	delayed free + cued recall (/16)	14	14	16	15	NA	NA
	**Anterograde visual memory**						
	Rey complex figure, memory (/36)	< centile 10	-	-	-	NA	NA
	**Language**						
	DO80 (/80)	-	-	60	-	NA	NA
	**Visuoconstructive praxies**						
	Rey complex figure, copy (/36)	25	12	13.5	-	NA	NA
**EEG**							
		Slow cerebral activity No epileptic or pseudo-periodic abnormalities	-	PSWC discharges during hyperpnea	unchanged	-	-

Cognitive domains are mentioned in bold characters. Abbreviations: *M* month, *NA* Not achievable by the patient, *“-”* not available, *“help”* the patient needed help to achieve the test, *MMSE* Mini Mental State Evaluation, *TMT* Trail Making Test, *FCSRT* Free and Cued Selective Recall Reminding Test, *FAB* Frontal Assessment Battery, *EEG* electroencephalogram, *PSWC* periodic sharp-wave complex

- Month 2: His condition worsened, and he was hospitalized in the Department of Neurology, in University Hospital, Toulouse, France. In the absence of sustained stimulation, the patient remained motionless and speechless, due to severe apathy. Falls occurred frequently (about once a day), due to altered postural reflexes. After marked stimulation, it was noticed that the patient was depressed but not melancholic or suicidal. Marked symmetric and axial parkinsonian syndrome did not improve after L-dopa challenge. Slight reflex myoclonus was noticed in the upper limbs. Ocular saccades were slow but not limited. Apraxia and executive dysfunctions had worsened. Detection of 14–3–3 protein performed from cerebrospinal fluid (CSF) sample by western-blot was positive, whereas cytology and biochemistry were normal. Structural cerebral MRI revealed fronto-temporal atrophy on visual assessment of T1 sequence, with no abnormality on diffusion weighted imaging (DWI). Ropinirole, and tianeptine treatments were replaced by citalopram, oxazepam and zopliclone.
- Month 6: Daily risperidone treatment was introduced due to delusions of persecution and rare visual hallucinations.
- Month 7: Parkinsonism did not deteriorate with the addition of neuroleptic agents. Delusions and hallucinations remained present, although less prominent, and cognitive functions kept worsening. Structural MRI was unchanged, and ^18^F-FDG-PET visual assessment showed severe hypometabolism in parietal regions.
- Month 10: The patient only experienced few hallucinations without delusions. Risperidone treatment was stopped but parkinsonian symptoms had worsened: he had severe dysarthria and was not able to walk without assistance because of his instability.

The patient had to be institutionalized 12 months after symptoms onset.

- Month 20: Axial parkinsonian symptoms had worsened: he had difficulties swallowing, severe dysarthria, freezing in narrow place and total loss of postural reflexes. He was bed-ridden most of the day. CSF 14–3–3 protein remained positive, while total-tau (238 pg/mL) and Aβ42 (511 pg/ml) levels were within the normal ranges (norm < 450 pg/mL and > 500 pg/mL, respectively).

MRI showed moderate striatal, brainstem, and bilateral fronto-temporo-parietal atrophy on visual assessment.

- Month 43: Parkinsonian symptoms were very severe, and myoclonus more pronounced. Important perseverations, imitations, and grasping were reported. Speech was unintelligible. Detection of 14–3–3 protein in CSF was negative.

The patient died one year later, aged 49, after a total duration of 55 months. Cerebral histopathological examination was performed.

### Imaging analyses

**Fig. 1:**
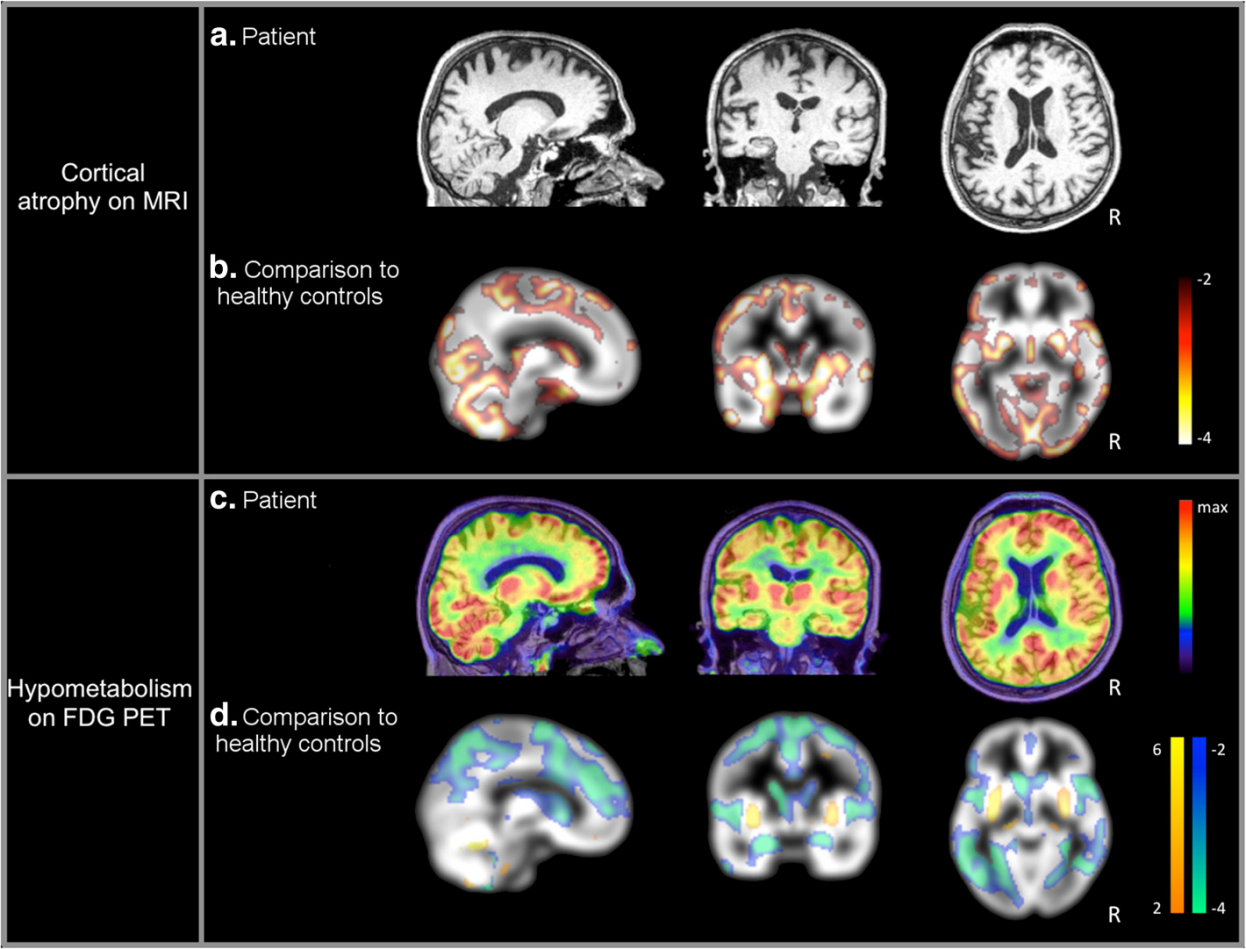
MRI and ^18^F-FDG-PET imaging. Patient’s MRI T1 sequence at M2 (**a**) and ^18^F-FDG–PETscan at M43 (**c**). Comparison between the patient and (**b**) age-matched healthy controls (*n* = 30) for grey matter density, (**d**) age-matched healthy controls (*n* = 23) for ^18^F-FDG uptake. Patient’s cortical atrophy below −2 standard deviations is displayed with a white to dark red scale. Patient’s hypometabolism below −2 standard deviations is displayed with a light green to dark blue scale while hypermetabolism is displayed with an orange to yellow scale. R = Right side

Grey matter density was assessed in our patient from his first T1 MRI sequence (Fig. 1a), and compared to the grey matter density of a group of 30 age-matched healthy controls (age = 44.9 ± 3.8) from the online database OASIS (http://www.oasis-brains.org/), using a whole brain z-score map. The ^18^F-FDG-PET scan from our patient (Fig. 1c) was also compared to another group of 23 age-matched healthy controls (age = 44.3 ± 9.4) using z-scores map (for details on the control population and imaging processing procedures, see Additional file 1). All z-scores below −2 or above 2 were considered as significant.

The patient showed global bilateral decrease of grey matter density affecting all cortical regions as well as subcortical structures and cerebellum (Fig. 1b and Additional file 2). Negative z-scores below −5 were found in parietal, temporal, but also frontal and insular cortices. Left hippocampus and bilateral thalami were spared. No z-score > 2 was found.

Regarding ^18^F-FDG-PET, the patient showed widespread significant bilateral hypometabolism, encompassing all cerebral lobes, most predominant in the parietal lobe (Fig. 1d and Additional file 2). Negative z-scores below −8 were found in parietal and medial temporal regions. No difference compared to controls was found in the thalamus, orbitofrontal regions, or the posterior cingulate. Hypermetabolism was found in the putamen.

### Histopathology

**Fig. 2:**
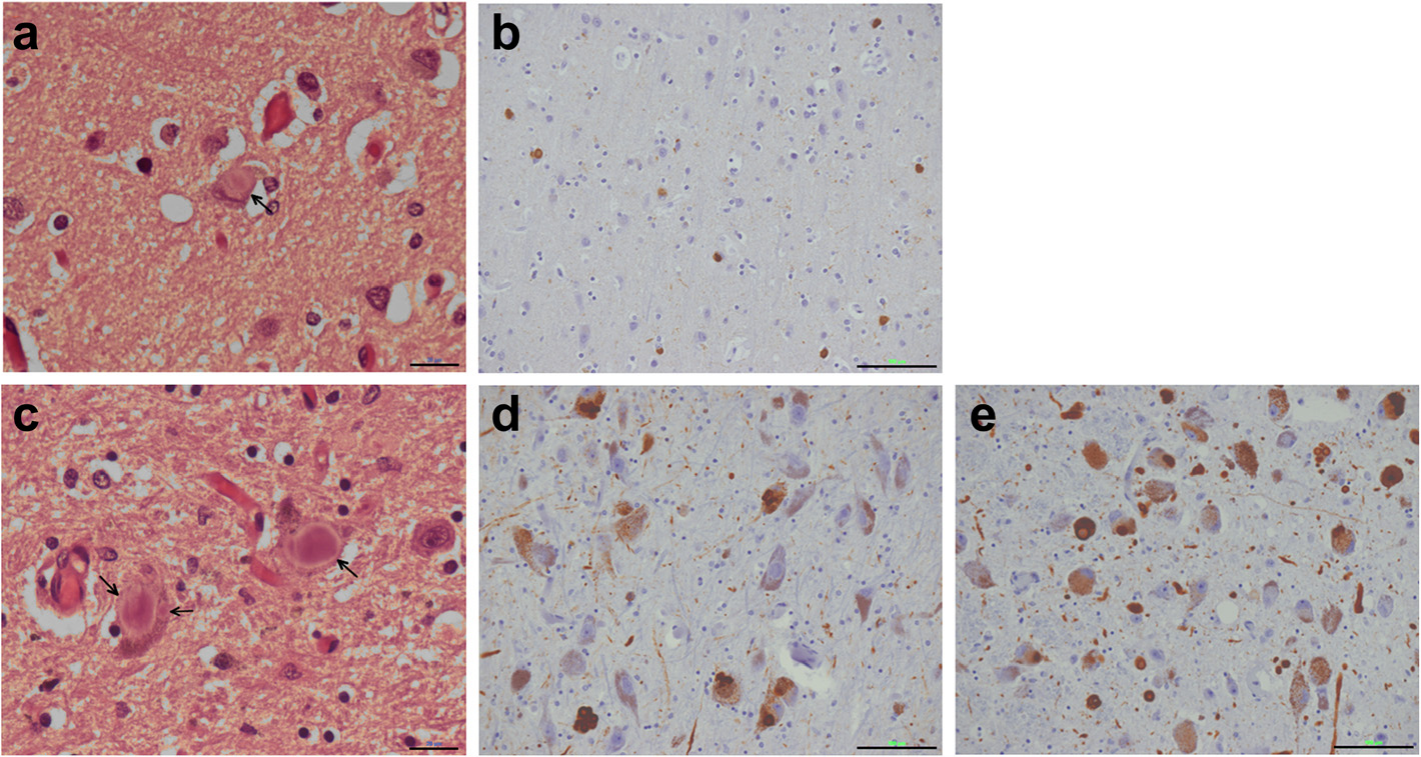
Post-mortem tissue staining. **a** and **c** Hematoxylin and eosin stained section of the frontal cortex (**a**) and the substantia nigra (**b**). Black arrows point at Lewy bodies in pigmented neurons. Bar scale = 20 μm. **b, d** and **e** Immuno-histochemistry with anti-alpha synuclein showing Lewy bodies in the frontal cortex (**c**), the substantia nigra (**d**) and the locus cœruleus (**e**)

Preliminary examination was carried out on hematoxylin-eosin stained paraffin sections, completed by immunohistochemistry with the following antibodies: anti-tau AT8 (Thermo Fisher Scientific, Illkirch, France), anti-amyloid-beta A4 (Dako, Trappes, France), anti-alpha-synuclein (Leica Biosystems, Newcastle, UK), and anti-prion protein 12 F10 (Spibio, Montigny Le Bretonneux, France). Macroscopic brain examination showed a marked palor of the substantia nigra, while anti-alpha-synuclein immunohistochemistry revealed numerous Lewy bodies and Lewy neurites in the frontal and temporal cortices, hippocampus, substantia nigra, locus cœruleus, as well as in the dorsal motor nucleus of the vagus, occipital and entorhinal cortices, and more rarely in the thalamus, caudate nucleus, putamen, and pallidum (Fig. 2). Spongiosis was absent and anti-prion protein immunohistochemistry was negative. A few AT8-positive neurofibrillary tangles were noticed in the hippocampus and the entorhinal cortex, whereas anti-amyloid-beta 4 staining revealed neither neocortical deposits nor amyloid angiopathy.

### Molecular genetics

*SNCA* gene (encoding the alpha-synuclein) mutations were investigated using bidirectional Sanger sequencing on an ABI 3730 automated sequencer (Applied Biosystem) and SeqScape v2.6 software (Applied Biosystems), and multiplex ligation-dependent probe amplification (MLPA, MRC-Holland). *PRNP* gene (encoding the prion protein) mutations were investigated using a previously described protocol [5]. Neither missense mutations nor multiplications were found in the *SNCA* gene (alpha-synuclein), and no mutation was identified in the *PRNP* gene (prion protein) either. The polymorphism at codon 129 was methionine-methionine.

## Conclusions

We report the case of a young patient with confirmed DLB that did not fulfil the revised clinical criteria [3] (see Table 2). Global cognitive impairment was indeed acute at onset, while progressive impairment is a mandatory requirement for DLB diagnosis. Moreover, no fluctuating cognition was observed during follow-up, while 75 % of DLB patients experience such fluctuations, which are a core feature of typical DLB [6]. Our patient also experienced slight visual hallucinations for one year, in a context of severe paranoid delusion without neuroleptic sensitivity, while delirium duration is usually short in DLB [7]. Moreover, hallucinations may also occur in CJD [8].

**Table 2:** Early diagnostic profile of the case according to the revised criteria for the clinical diagnosis of dementia with Lewy bodies (DLB) – adapted from McKeith et al., Neurology 2005 [3]

	Dementia with Lewy bodies	Creutzfeldt-Jakob disease	Case reported
Central feature			
Cognitive decline	Progressive, insidious onset	Acute or rapidly progressive	Acute
Prominent or persistent memory impairment	Not early: usually evident with progression	Early	Early
Prominent deficits of attention, executive function, and visuospatial ability	Yes	No (global dementia)	No (global cognitive impairment)
Core features			
Fluctuating cognition	Yes	No	No
Recurrent visual hallucination	Yes	Yes	No (mainly delirious with few hallucinations)
Spontaneous features of parkinsonism	Yes	Yes	Yes
Suggestive features			
REM sleep behavior disorder	Yes	No	Not reported by the wife
Severe neuroleptic sensitivity	Yes	No	No
Low dopamine uptake in basal ganglia on imaging	Yes	Very rare (one case reported [22])	Not performed (the case was parkinsonian)
Supportive features (Commonly present in DLB but not proven to have diagnostic specificity)			
Repeated falls and syncope	Yes	No	Yes
Transient loss of consciousness	Yes	No	No
Severe autonomic dysfunction	Yes	No	No
Hallucinations in other modalities	Yes	No	No
Systematized delusions	Yes	No	Yes
Depression	Yes	Yes	Yes
Relative preservation of medial temporal lobe structures on MRI	Yes	Yes	Yes
Generalized low uptake on PET perfusion scan with reduced occipital activity	Yes	No	Extensive hypometabolism
Prominent slow wave activity on EEG with temporal lobe transient sharp waves	Yes	No (biphasic and triphasic periodic complex)	Slow but without temporal lobe transient sharp waves
Other			
Mean age of onset (years)	75	70	42
Mean duration (years)	7	0.5	5
Detection of 14.3.3 protein	Rarely (3 cases reported)	Yes	Yes

*Probable DLB* Two core features or one core feature and one or more suggestive features, *Possible DLB* Up to one core feature and one or more suggestive features

Features for Creutzfeldt-Jakob disease are adapted from the MRI-CJD Consortium criteria for sporadic Creutzfeldt–Jakob disease [8]

The patient’s clinical presentation was similar to the cognitive subtype of CJD described by Puoti et al. [9], with acute dementia, confusion and cortical visual disturbance, extrapyramidal signs and myoclonus. Although MRI did not show diffusion abnormalities, such clinical presentation and positive 14.3.3 protein detection were consistent with CJD diagnosis [8]. Cases of DLB are reported in neuropathological series of suspected CJD [4, 10–12]. But in these series, patients are at least 60 years-old; symptomatic progression is rapid and leads to death within one to two years. Despite an acute onset, our 44-year-old patient showed a clinical progression and disease duration similar to usual DLB [13], and no longer fulfilled the clinical criteria for sporadic CJD disease [8] two years later.

Interestingly, patients suspected for CJD during lifetime but with confirmed DLB at autopsy often share myoclonus, pyramidal and extrapyramidal symptoms [10]. While fluctuating cognition and repeated falls are rare, hallucinations and akinetic mutism are variable among the series. When performed, 14–3–3 protein detection is negative in most cases, except for Van Everbroeck and colleagues who reported rare 14–3–3 protein positivity (less than 20 % of definite DLB) [14]. Of note, a recent study presented tau protein levels as a better marker than 14–3–3 protein in the diagnosis of CJD [15]. After 2 years of clinical evolution, CSF total tau level was within the normal range, confirming the hypothesis that our patient had no CJD.

As observed in our case, none of the previously reported confirmed LBD patients with suspicion of CJD had cerebellar symptoms, or hypersignal on MRI, but most of them had diffuse cortical atrophy [10]. Regarding our patient, comparison to a control population revealed a wide bilateral atrophy involving all cortical regions, as well as subcortical nuclei such as the putamen and the pallidum. Decrease of grey matter density in the insula and the putamen has been recently reported as a characteristic pattern of atrophy in patients with DLB [16]. This pattern should also include lateral temporal atrophy, but we reported a broader pattern of atrophy, including other cortical regions. This extended atrophy suggests an advanced stage of the pathology in the patient’s brain, although the MRI used was performed soon after clinical onset.

The patient also presented with widespread cortical hypometabolism, sparing orbitofrontal regions as well as posterior cingulate and thalamus. Interestingly, we found bilateral hypermetabolism in the posterior part of the putamen. To our knowledge, this is the first report of striatal hypermetabolism in DLB [17]. Compensatory mechanisms may explain this finding, but further investigations are required.

While mean age of onset for DLB is usually 75 years old, ranging from 50 to 80, DLB can also be an uncommon cause of dementia in young patients [18]. Few patients have been reported with DLB before 50 years-old [2]. Most of them were reported in Japan [19], although they did not fulfill the revised criteria for DLB, and may not have had pure DLB [20]. Because our case was sporadic and because parkinsonism was the core clinical feature, molecular genetic analysis was limited to the alpha synuclein and prion protein genes. However, other genetic variations were also reported in DLB [21]. In this case, the implication of these other genes is unlikely, considering the pathological findings of Lewy bodies and Lewy neurites without beta amyloid plaques or widespread neurofibrillary tangles.

In summary, our case illustrates that DLB can occur in young patients with an atypical and severe clinical presentation, which can first be mistaken for CJD. Laboratory and neuroimaging investigations are useful to exclude systemic and pharmacological causes of delirium and cognitive impairment, but remain insufficient to allow an accurate diagnostic of DLB, particularly in young patients. Specific markers to support such diagnosis are needed.

## Abbreviations

CJD, Creutzfeldt-Jakob Disease; CSF, Cerebrospinal Fluid; DLB, Dementia with Lewy Bodies; FDG, Fluorodeoxyglucose; MRI, Magnetic Resonance Imaging; PET, Positron Emission Tomography

## Additional files

Additional file 1: Details on imaging methodological procedures. (DOC 23 kb) Additional file 2: Imaging regional quantification. The table displays z-scores from quantitative comparison of ^18^F-FDG uptake and grey matter density between the patient and age-matched control groups. (DOC 92 kb)
