# Supplementary material for: Case report of Lewy body disease mimicking Creutzfeldt-Jakob disease in a 44-year-old man
Source: BMC Neurol. 2016 Jul 30;16:122. doi: 10.1186/s12883-016-0643-y (PMC4967506; doi:10.1186/s12883-016-0643-y)
Supplement: Additional file 1: — Details on imaging methodological procedures. (DOC 23 kb) [file 12883_2016_643_MOESM1_ESM.doc]

**Supplementary File 1**: **Details on imaging methodological procedures**

MRI: Grey matter density in our patient was assessed from the first T1 MRI sequence (M2), and was compared to the grey matter density of a control group using a whole brain z-score map. This z-score map was created using SPM8 software (http://www.fil.ion.ucl.ac.uk/spm/software/spm8/) as follow: all T1 images were segmented for grey matter, and the resulting modulated probability maps were registered into MNI space and smoothed (8x8x8). Finally, both the average and standard deviation (SD) maps were created for the control group, and a z-score map was created for the patient.

All z-scores below -2 or above 2 were considered as significant (negative scores corresponding to regions with decreased grey matter density and positive scores to those with increased density).

FDG: The patient’s ^18^F-FDG scan performed at M47 was also compared to a group of controls in the form of a whole brain z-scores map using SPM8 software. The controls were free from neurological/psychiatric disease and cognitive complaints, and had a normal brain MRI. All ^18^F-FDG scans were registered into MNI space and smoothed (8x8x8). A binarized grey matter probabilistic map from SPM8 was used as inclusive mask and subsequently applied to all scans. Cortical uptake was quantified using the grey matter of the cerebellum as reference region. Finally, both the average and standard deviation (SD) maps were created for the control group, and a z-score map was created for the patient.

All z-scores below -2 or above 2 were considered as significant (negative scores corresponding to hypometabolic regions and positive scores to hypermetabolic regions).
