# Supplementary material for: Case report of Lewy body disease mimicking Creutzfeldt-Jakob disease in a 44-year-old man
Source: BMC Neurol. 2016 Jul 30;16:122. doi: 10.1186/s12883-016-0643-y (PMC4967506; doi:10.1186/s12883-016-0643-y)
Supplement: Additional file 2: — Imaging regional quantification. The table displays z-scores from quantitative comparison of 18F-FDG uptake and grey matter density between the patient and age-matched control groups. (DOC 92 kb) [file 12883_2016_643_MOESM2_ESM.doc]

|  |  | ^18^F-FDG PET | | MRI T1 | |
| --- | --- | --- | --- | --- | --- |
|  |  | LEFT | RIGHT | LEFT | RIGHT |
| **FRONTAL** | Precentral | -5.54 | -4.56 | -5.77 | -5.24 |
|  | Frontal_Sup | -7.05 | -5.21 | -5.31 | -3.83 |
|  | Frontal_Sup_Orb | ns | ns | -3.17 | -2.96 |
|  | Frontal_Mid | -4.84 | -5.63 | -5.63 | -4.66 |
|  | Frontal_Mid_Orb | -2.45 | -2.24 | -3.73 | -3.43 |
|  | Frontal_Inf_Oper | -3.44 | -5.01 | -4.08 | -4.39 |
|  | Frontal_Inf_Tr | -3.87 | -3.04 | -4.28 | -3.82 |
|  | Frontal_Inf_Orb | -3.93 | -4.02 | -4.16 | -3.42 |
|  | Rolandic_Oper | -5.50 | -5.22 | -3.62 | -3.05 |
|  | Supp_Motor_Area | -6.53 | -5.59 | -4.90 | -4.95 |
|  | Olfactory | -3.90 | -3.16 | -3.66 | -3.13 |
|  | Frontal_Sup_Medial | -5.71 | -4.81 | -4.44 | -3.97 |
|  | Frontal_Med_Orb | -3.53 | -4.02 | -2.64 | -3.14 |
|  | Rectus | -3.46 | -2.28 | -2.98 | -3.22 |
|  | Cingulum_Ant | -5.71 | -5.59 | -3.00 | -3.21 |
| **INSULA** | Insula | -5.23 | -5.87 | -4.01 | -5.01 |
| **PARIETAL** | Cingulum_Mid | -5.95 | -5.44 | -4.51 | -3.91 |
|  | Cingulum_Post | -2.36 | ns | -1.95 | -2.83 |
|  | Postcentral | -5.93 | -6.94 | -5.22 | -5.35 |
|  | Parietal_Sup | -8.21 | -8.35 | -4.68 | -5.18 |
|  | Parietal_Inf | -8.29 | -4.86 | -4.55 | -4.06 |
|  | SupraMarginal | -6.43 | -5.56 | -4.93 | -4.26 |
|  | Angular | -6.89 | -7.21 | -3.89 | -5.56 |
|  | Precuneus | -7.53 | -5.56 | -4.61 | -4.18 |
|  | Paracentral_Lobule | -4.58 | -4.47 | -4.98 | -4.34 |
| **OCCIPITAL** | Occipital_Sup | -5.27 | -5.58 | -3.85 | -3.91 |
|  | Occipital_Mid | -7.53 | -5.27 | -5.56 | -4.34 |
|  | Occipital_Inf | -5.95 | -3.98 | -4.28 | -4.40 |
|  | Calcarine | -4.98 | -3.58 | -4.48 | -4.22 |
|  | Cuneus | -5.33 | -3.59 | -4.67 | -4.78 |
|  | Lingual | -6.27 | -4.27 | -4.21 | -4.89 |
| **TEMPORAL** | Hippocampus | -5.72 | -4.52 | -4.28 | ns |
|  | ParaHippocampal | -6.34 | -4.81 | -4.26 | -3.67 |
|  | Amygdala | -6.22 | -4.97 | -4.55 | -3.92 |
|  | Fusiform | -6.06 | -5.63 | -3.87 | -4.06 |
|  | Heschl | -6.27 | -4.92 | -3.43 | -3.01 |
|  | Temporal_Sup | -6.71 | -7.73 | -4.59 | -5.50 |
|  | Temporal_Pole_Sup | -3.92 | -5.30 | -4.62 | -5.46 |
|  | Temporal_Mid | -6.95 | -7.16 | -3.90 | -4.08 |
|  | Temporal_Pole_Mid | -4.31 | -1.43 | -4.14 | -2.29 |
|  | Temporal_Inf | -9.41 | -5.43 | -4.98 | -3.56 |
| **SUBCORT** | Caudate | -7.47 | -4.31 | -3.26 | -3.58 |
|  | Putamen | 6.61 | 4.83 | -4.72 | -4.90 |
|  | Pallidum | -4.94 | -3.05 | -4.82 | -4.90 |
|  | Thalamus | ns | ns | ns | ns |

**Supplementary File 2**: Imaging regional quantification. The table displays z-scores from quantitative comparison of ^18^F-FDG uptake and grey matter density between the patient and age-matched control groups. Values below -2 or above 2 are considered significant and displayed in the table. “ns” = not significant.
